# The use of laser-assisted cart positioning significantly reduces the docking time of multimodular robotic systems

**DOI:** 10.1007/s11701-024-02196-y

**Published:** 2025-01-06

**Authors:** Martin Baunacke, Christopher Hirtsiefer, Roman Herout, Sherif Mehralivand, Susanne Oelkers, Oliver Kaske, Claudia Franz, Christian Thomas

**Affiliations:** 1 Department of Urology, Medical Faculty Carl Gustav Carus, TU Dresden, Fetscherstr. 74, 01307 Dresden, Germany; 2 Department of Medical Technology, Medical Faculty Carl Gustav Carus, TU Dresden, Dresden, Germany

**Keywords:** Hugo RAS, Multimodular robotic systems, Docking, Radical prostatectomy

## Abstract

The Hugo RAS system is characterized by its multimodular design, which leads to an increased docking effort. Exact data for docking time and the learning curve is missing. We describe for the first time the use of a laser-guided cart positioning to reduce the docking time. In this prospective monocentric study, the docking time was evalutated for a consecutive series of pelvic surgeries with the Hugo RAS system. In a subgroup, a cross-line laser was adapted at the cart for positioning using fix points at the ceiling. The medical personnel were classified as “inexperienced” with ≤ 5 consecutive dockings and as “experienced” with > 5 consecutive dockings. From 10/2023 to 08/2024, 82 procedures were performed with the Hugo RAS. For the evaluation 75 procedures could be considered. The mean docking time was 7.6 ± 3.5 min. There was a reduction in docking time from 13.5 ± 3.7 min in the first 5 procedures to 4.4 ± 0.9 min in the last 5 procedures (*p* < 0.001). Docking with laser (*n* = 45) was faster than without laser (*n* = 30) (6.2 ± 2.5 vs. 9.8 ± 3.7 min, *p* < 0.001). Faster docking time was observed with inexperienced surgical nursing staff with laser than without laser (10.4 ± 3.7 vs. 5.4 ± 1.4 min; *p* < 0.001). With experienced nursing staff, the laser had no influence (6.6 ± 1.3 vs. 6.7 ± 2.9 min; *p* = 0.9). As a reference docking time for daVinci Xi procedures was 2.4 ± 1.7 min (*n* = 5). Laser-guided cart positioning has a significant impact on docking time, especially for unexperienced medical personnel. Especially in the times of experienced staff shortage, laser-guided cart positioning can save operating time.

## Introduction

The advent of robotic surgical systems has revolutionized urologic surgery within the last quarter of the century. Especially for reconstructive urooncology and malformations of the urinary tract in adults, robotic surgical systems have thrusted open surgery in the background in daily clinical practice [1, 2]. Moreover, its use is more and more advocated by the public to minimalize invasiveness in surgery [3–6]. On the other hand, only the acquisition and maintenance costs for robotic systems but also its handling in daily clinical practice is a challenge for the medical personnel and hospital operators [7, 8]. Within the last two decades the Da Vinci robotic platforms have prevailed the market and display the state of the art in robotic urology. Based on a single patient cart, multiport docking can be easily performed especially with the fully automated Xi system. However, external collisions of robotic arms are still an issue especially in earlier Da Vinci robots. Arm collision demands modification of the robotic arm axis and in worst case new port-placement resulting in longer operation time. Theoretically, multi cart robotic systems have the advantage of individualized cart placement and configuration of the robotic arm axis, which should result in collision-free motion. However, proper positioning of the arm-carts is crucial and can result in a prolonged docking time, especially for unexperienced medical personnel. Currently, the HUGO RAS robotic system from Medtronic is the mostly used multi-cart telemanipulator in urology [9]. While cart placement for sufficient system configuration is precisely described in the setup guide, practical implementation in daily routine is crucial and demands an exact positioning of each cart. Marking the operation room floor with tape is an option for exact cart positioning; however, must be seen controversial regarding hygienic standards. We hypothesize that a laser-guided cart placement significantly reduced docking time for multi-cart robotic systems without affecting hygienic standards.

## Materials and methods

**Fig. 1:**
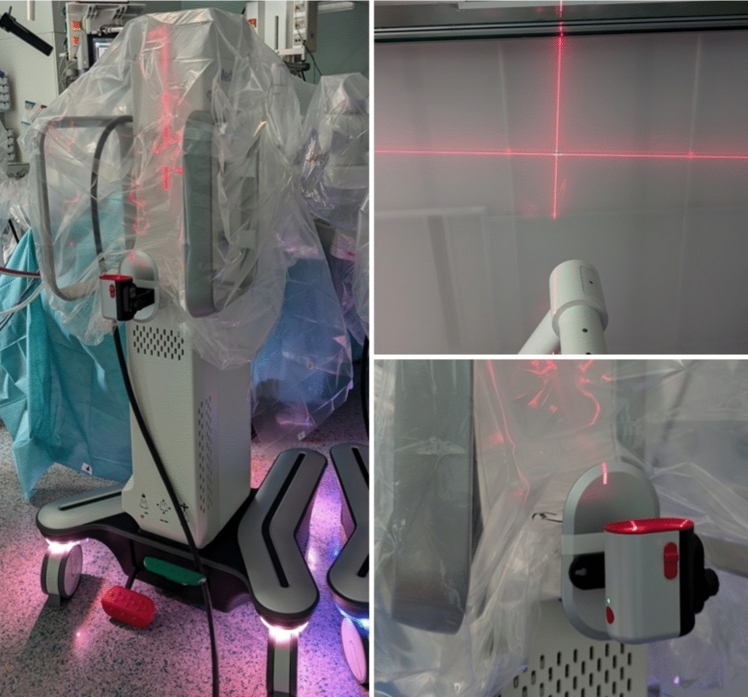
Kart with laser and ceiling marking

In this prospective single-center study we recorded docking times with the Hugo RAS our department from October 2023 until August 2024. The docking time was defined from the end of trocar placement to the start of the console surgery. We included all transperitoneal interventions in Trendelenburg position with the usage of 4 arms and the same trocar placement. At the start of docking, all 4 carts are at a distance from the operating table. The tilt angles are already preset. The docking angles are set during docking (Arm 1: 105°, Arm 2: 130°, Arm 3: 175°, Arm 4: 220°). Karts are moved by one nurse, arm and instrument placement is performed by two surgeons. For laser assistance we used a cross-line laser, which was attached at the back of the carts in a specific position and fixed with a magnet. The laser cross shines on the ceiling, on which there are markings for the four kart positions (Fig. 1). To record the docking time, medical personnel with ≤ 5 consecutive dockings was designated as “inexperienced” and with > 5 consecutive dockings as “experienced”. In detail, medical personnel were subgrouped in scrub nurses and urology residents who moved the carts. Due to extensive experience in cart-docking, console surgeons were excluded. Reference docking time was determined by five times docking with the daVinci Xi system. These dockings were carried out by personnel considered to be experienced. Data were analyzed using the t-test und linear regression. Propensity score matching was used to exclude influence of experience of the bedside surgeon on docking times depending on the experience of the nurse. It was also used exclude influence of nurse experience and laser usage on docking times depending on the experience of the bedside surgeon. *P* ≤ 0.05 was considered to indicate significance. All calculations were performed with “IBM SPSS Statistics 28” (Armonk, New York, USA). The study was approved by the local ethics committee (BO-EK-273062023). Ethical approval involved consultation with a data protection officer, ensuring secure data handling.

## Results

### Collective

From 10/2023 to 08/2024, 82 robot-assisted transperitoneal procedures were performed with the Hugo RAS. For the evaluation of the docking time, 75 procedures were included: 69 radical prostatectomies, 5 simple prostatectomies, 1 resection of a vesicovaginal fistula. Seven and three procedures were not included due to cart malfunction and incomplete documentation, respectively. The five reference surgeries with daVinci were radical prostatectomies. A total of 4 console surgeons, 7 bedside surgeons and 12 scrub nurses were involved in all 75 procedures.

### Docking time

**Fig. 2:**
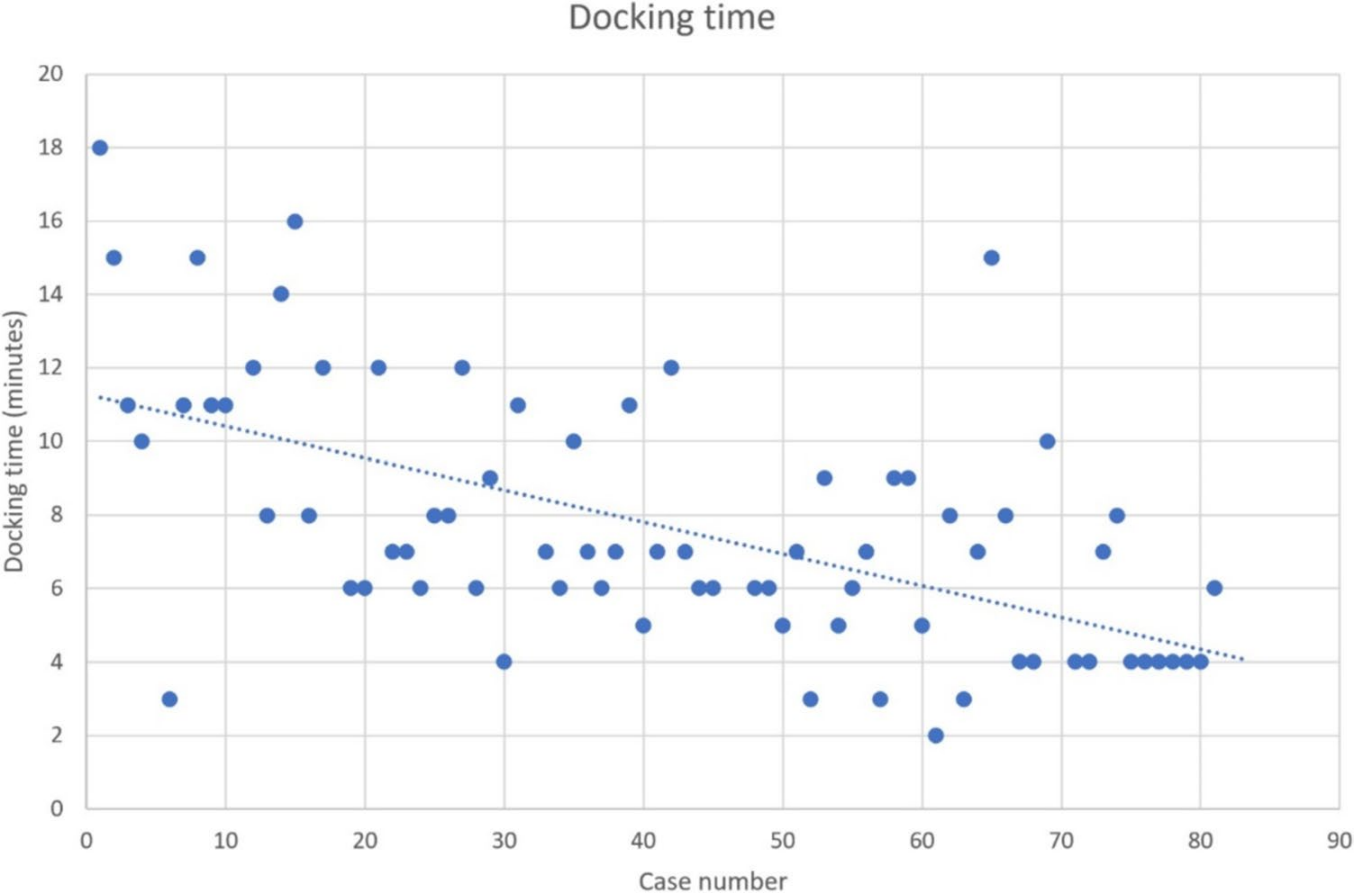
Docking time with Hugo RAS from the first to the 82nd case (*n* = 75)

The average docking time with Hugo RAS was 7.6 ± 3.5 min (13 [10, 18] min). There was a reduction in docking time from 13.5 ± 3.7 min in the first 5 interventions in October 2023 to 4.4 ± 0.9 min (4 (46) min) in the last 5 interventions in September 2024 (p < 0.001) (Fig. 2). As a reference, the time of five dockings with daVinci system was 2,4 ± 1,7 min (2 (15) min) (*n* = 5). Without laser assistance docking time was faster with experienced nurses than with inexperienced nurses (10.4 ± 3.7 vs. 6.6 ± 1.3 min; *p* = 0.001). There was no significant difference depending on the experience of the bedside surgeon (11.2 ± 4.5 vs. 8.8 ± 2.7; *p* = 0.08).

### Docking time with laser assistance

Docking with laser (*n* = 45) was shorter than without laser (*n* = 30) (6.2 ± 2.5 vs. 9.8 ± 3.7 min, *p* < 0.001). There was a significantly faster docking time with inexperienced OR nursing with laser than without laser (10.4 ± 3.7 vs. 5.4 ± 1.4 min; *p* < 0.001). Experienced nursing staff showed no influence of the laser (6.6 ± 1.3 vs. 6.7 ± 2.9 min; *p* = 0.9). There was also a significantly faster docking time with inexperienced bedside surgeon with laser than without laser (11.2 ± 4.5 vs. 6.5 ± 3.0 min; *p* < 0.001) and with experienced bedside surgeon with laser than without laser (11.2 ± 4.5 vs. 6.5 ± 3.0 min; *p* < 0.001) (Table 1).

**Table 1:** Univariate analysis of docking time depending on laser usage and experience of nurse and bedside surgeon (*n* = 75)

	All procedures (*n* = 75)	Procedures without laser (*n* = 30)	Procedures with laser (*n* = 45)	*p* value
Total docking time (min)	7.6 ± 3.5 7 (2–18)	9.8 ± 3.7 10.5 (3–18)	6.2 ± 2.5 6 (2–15)	** < 0.001**
Docking time by nurse experience (min)				
Inexperienced (1–5 procedures)	8.3 ± 3.9 7 (3–18)	10.4 ± 3.7 11 (3–18)	5.4 ± 1.4 6 (3–7)	** < 0.001**
Experienced (≥ 6 procedures)	6.7 ± 2.7 6.5 (2–15)	6.6 ± 1.3 6 (5–8)	6.7 ± 2.9 7 (2–15)	0.9
Docking time by bedside surgeon experience (min)				
Inexperienced (1–5 procedures)	8.7 ± 4.4 8 (3–18)	11.2 ± 4.5 11 (3–18)	6.5 ± 3.0 6 (4–15)	**0.004**
Experienced (≥ 6 procedures)	7.0 ± 2.7 7 (2–12)	8.8 ± 2.7 8 (5–12)	6.0 ± 2.3 6 (2–11)	**0.001**

Bold values are statistically significant (*p* < 0.05)

### Propensity score matching docking (PSM) time with laser assistance

The PSM matching for experience of the bedside surgeon (*n* = 64) confirms the faster docking time for inexperienced nurses with laser than without laser (9.9 ± 3.1 vs. 5.5 ± 1.6 min; *p* < 0.001) and the lack of influence of the laser for experienced nurses (6.6 ± 1.3 vs. 6.7 ± 2.9 min; *p* = 0.9) (Table 2). The PSM matching for nurse experience and laser usage (*n* = 56) shows that there is no influence of the bedside surgeon experience on the docking time (8.7 ± 4.4 vs. 8.2 ± 2.4 min, *p* = 0.6) (Table 3).

**Table 2:** Propensity score matching (matching bedside surgeon experience) analysing of docking time depending on laser usage and experience of nurse (*n* = 64)

	All procedures (*n* = 64)	Procedures without laser (*n* = 25)	Procedures with laser (*n* = 39)	*p* value
Total docking time (min)	7.5 ± 3.1 7 (2–15)	9.2 ± 3.1 9 (3–15)	6.3 ± 2.6 6 (2–15)	** < 0.001**
Docking time by nurse experience (min)				
Inexperienced (1–5 procedures)	8.3 ± 3.4 7 (3–15)	9.9 ± 3.1 11 (3–15)	5.5 ± 1.6 6 (3–7)	** < 0.001**
Experienced (≥ 6 procedures)	6.7 ± 2.7 6.5 (2–15)	6.6 ± 1.3 6 (5–8)	6.7 ± 2.9 7 (2–15)	0.9

Bold values are statistically significant (*p* < 0.05)

**Table 3:** Propensity score matching (matching nurse experience and laser usage) analysing of docking time depending on bedside surgeon experience (*n* = 56)

	All procedures (*n* = 56)	Inexperienced bedside surgeon (*n* = 28)	Experienced bedside surgeon (*n* = 28)	*p* value
Total docking time (min)	8.4 ± 3.5 7 (3–18)	8.7 ± 4.4 8 (3–18)	8.2 ± 2.4 7 (5–12)	0.6

## Discussion

The docking time with Hugo RAS can be significantly reduced with increasing experience (13.5 ± 3.7 min vs. 4.4 ± 0.9 min (*p* < 0.001). Despite its impact, multi-cart docking time is still longer than with the daVinci Xi system (2,4 ± 1,7 min). The introduction of a laser-guided cart positioning will lead to shorter docking times (6.2 ± 2.5 vs. 9.8 ± 3.7 min, *p* < 0.001). This effect is particularly noticeable in inexperienced nurses (5.4 ± 1.4 vs. 10.4 ± 3.7 min; *p* < 0.001). The experience of the bedside surgeon plays no role in the docking process.

When considering the acquisition of a new robotic-assisted system, the question arises as to whether to fall back on the established da Vinci system, with which the vast majority of robot surgeons already have experience, or to embrace a new system. Several studies already have already shown the reliability of the Hugo RAS system [10, 11]. Thus, there were no concerns about being able to continue the surgical quality with this system as well. However, most concerns are related to the handling and the time effort of docking with multi-cart systems. In our study, we show that the initial docking time with the multi-port system was about five times longer than with the DaVinci Xi-system (13.5 ± 3.7 min vs. 2,4 ± 1,7 min). Even after establishment with experienced personnel, the docking time is still about two times longer (4.4 ± 0.9 min). Another group from Italy with an experience of 132 multi-cart cases demonstrated an average docking-time of 10 ± 2 min [12]. This corresponds to our average docking time without a laser including experienced and inexperienced personnel (9.8 ± 3.7 min). Interestingly, an Italian group just using three carts in 86 RARP was able to perform cart-docking in a mean time of 4 min [13]. A prospective single-center study comparing DaVinci and Hugo RAS with 75 cases in each procedure showed longer docking times with Hugo RAS (18.62 min) and DaVinci (10.45 min) [14]. These data were confirmed by another group by comparing 50 Hugo RAS vs. 50 DaVinci RARP (11 min vs. 6 min, respectively) [15]. Also a systematic review analysed docking time in three comparative studies. They showed a better docking time for DaVinci RARP with a mean difference of 6.43 min [16]. In this context, laser-guided cart positioning will have a significant time difference in docking time. To notice, in our study the average docking time with laser-guidance was 6.2 ± 2.5 min and herewith faster than all above mentioned studies. Interestingly, we were not able to show a difference in docking time between experienced nurses with and without laser-assistance (6.6 ± 1.3 vs. 6.7 ± 2.9 min; *p* = 0.9), reflecting the huge impact of experience over time [17].

In addition to the cart-movement, another aspect of docking is the direct docking of the arms to the trocars with the appropriate adjustment of the angles and the introduction of the instruments. At our clinic, this process is carried out by the console surgeon and the bedside surgeon. It starts after the first arm is approached and is carried out in parallel with the approach of the other three arms. In matching of nurse experience and laser usage there is no difference in docking time between inexperienced and experienced bedside surgeons (8.7 ± 4.4 vs. 8.2 ± 2.4 min, *p* = 0.6). This underlines the importance of nurse experience and the cart movement itself in docking process.

Console time still has the most time-consumable impact in RARP. Interentially, the console surgeon plays a particularly important role. With increasing experience, the operating time can be significantly reduced [18]. The current Hugo RAS studies show a console time between 138 and 242 min [12, 14, 15]. In this context, the docking time seems rather negligible. But in an experienced clinic with experienced console surgeons, the docking time is gaining importance in reduction of OR time. Such optimizations can be particularly relevant in the context of an aging population, which is leading to an increasing number of patients on the one hand, and a growing shortage of nursing staff due to a lack of new recruits on the other [5, 19]. The totality of process optimizations in robotic-assisted surgery can determine whether two or three consecutive RARP procedures can be performed in one operating room.

Our study has several limitations. There is the bias due to lack of blinding in usage of the laser. Even though the staff knew that the laser was being used to accelerate the docking process, the docking time was not the sole focus of the parallel time study during the operation, as other times were also recorded. All staff which was introduced to Hugo RAS was already experienced in robotic surgery with the DaVinci system. So far, we do not have any data from personnel who is completely unexperienced in the use of robotic-assisted systems. We also did not randomize the procedures for laser or no laser. The laser-guided positioning was introduced after the first 32 multi-cart procedures. Nevertheless, it was possible to compare these data, since scrub nurses unexperienced in multi-cart robotics were introduced step by step in the course of these 75 interventions and the laser application was also not used continuously after the first 32 interventions either.

In summary, we show for the first time that laser-guided positioning of multi-cart robotic systems significantly improves docking time, which translates in cost-reduction over time. Unexperienced scrub nurses benefitted most from this easy applicable guiding system. Additionally, we demonstrate that docking-time mostly depends on experience of scrub nurses and in a lesser extent on surgeons. In all Hugo RAS publications available so far, guiding options for optimal cart positioning, were not mentioned. To our knowledge, several clinics using this system put tapes on the floor of the operating room for exact cart positioning. In our opinion this option is suboptimal especially regarding hygienic aspects and obstructed view by the cart itself. The markings on the ceiling and the laser positioning are hygienically safe, more visible and do not wear off. Therefore, we hope that this study will provide other clinics an easy applicable and low-cost option for accelerated cart-docking. To get further insight in the topic, studies that randomly assess cart positioning with floor marking, laser guidance, and without assistance are needed.

## Conclusions

As cart positioning is crucial, so far multi-cart docking is more time consumable than single-cart docking. For multimodular robotic systems, laser-guidance significantly improves docking time, which translates in cost-reduction over time.

## Data Availability

No datasets were generated or analysed during the current study.
